# A selective and easily recyclable dimer based on a calix[4]pyrrole derivative for the removal of mercury(ii) from water†

**DOI:** 10.1039/c9ra09911e

**Published:** 2020-01-16

**Authors:** Angela F. Danil de Namor, Salman Alharthi, Brendan Howlin, Nawal Al Hakawati

**Affiliations:** Laboratory of Thermochemistry, Department of Chemistry, University of Surrey Guildford Surrey GU2 7XH UK a.danil-de-namor@surrey.ac.uk +44(0)-1483 689581 +44(0)-7757147701; Instituto Nacional de Tecnologia Industrial, Ministry of Production Argentina

## Abstract

A recyclable mercury(ii) selective dimer based on a calix[4]pyrrole derivative has been synthesised and characterised by mass and FT-IR spectrometry, Scanning Electron Microscopy (SEM) and Energy Dispersive X-ray (EDX). Information regarding the ability of the dimer to interact with metal cations was obtained from FTIR and SEM-EDX analyses. A striking feature of micrographs of the loaded dimer is the change of morphology with the cation. Based on these results, optimal conditions for removing cations from water were assessed under different experimental conditions. Results obtained demonstrate that the removal process is fast. Capacity values and selectivity factors show that the dimer is selective for Hg(ii) in single and multiple component metal solutions relative to other cations. Single-ion transfer Gibbs energies from water to a solvent containing common functionalities to those of the dimer were used to assess the counter-ion effect on the removal process. Agreement is found between these data and energy calculations derived from molecular simulation studies. Studies on polluted water in the presence of normal water components in addition to toxic metal cations are reported. Further experimental work on wastewater from the mining industry is in progress.

## Abstract 2

A recyclable mercury(ii) selective dimer based on a calix[4]pyrrole derivative has been synthesised and characterised by mass and FT-IR spectrometry, Scanning Electron Microscopy (SEM) and Energy Dispersive X-ray (EDX).

## Graphical abstract

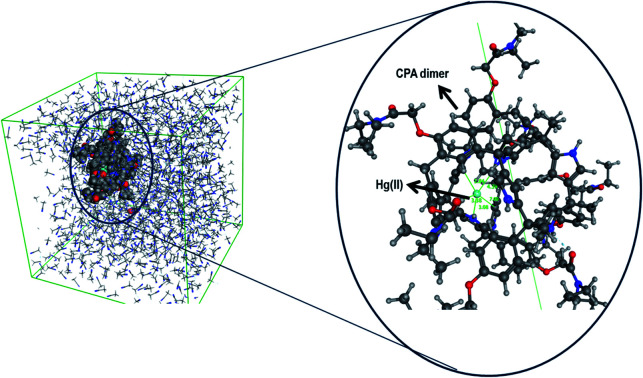

## Introduction

Contamination of the ecosystem by mercury is a topic of priority due to the implications on human health and the environment^1–7^ as emphasised in an excellent report^8^ in which all aspects associated with mercury pollution are addressed. This is a research area not only relevant to the developing World but also to highly industrialised Countries.^9–17^ Mercury contamination is a global problem to an extent that the Minamata Convention has been ratified by 128 Countries.^18^ Although this convention aims to reduce/eliminate the use of mercury in industrial and medical processes it also encourages research on developing monitoring systems and technologies for the removal of mercury from contaminated sources. Physical and chemical methods such as precipitation/co-precipitation, ion exchange, membrane filtration have been mostly used for targeting heavy metals in general. Advantages and limitations of these approaches have been extensively reviewed in the literature.^19^ However these are often non-selective.

Most approaches for removing mercury from the ecosystem are focused on bioremediation processes^19–25^ which is a low cost technology. As far as the uptake of metals by plants is concerned, current species identified as natural carriers for removing heavy metals are phytochelatins,^26^ CPx-type ATPases,^27,28^ ZIP transporters,^29^ natural resistance-associated macrophage proteins,^30,31^ organic and amino acids^32^ but an essential requirement for its success is the choice of the natural carrier and this is by no means a straightforward selection. Additionally they require large areas, maintenance and operational costs. In recent years a number of papers have been published on new approaches for removing mercury from water using functionalised silica sorbents based on mesoporous materials,^33^ nanoparticles^34–37^ and chelating agents.^38^

Supramolecular chemistry^39^ offers the possibility of producing highly selective receptors which can mimic some of the receptors present in living systems^40^ for removing metal ions from water with the additional advantage that these can be easily recyclable, an important issue for the commercialisation of any technology, within this context calixpyrroles (products of the condensation of pyrrole and ketones in acid medium) possess properties of great interest, not the least as extracting agents for removing pollutants from the environment. However, most of the work dealing with the use of macrocycles for extraction processes have been centred on liquid–liquid extraction processes due to the solubility of the monomer in organic medium. While initial contributions to calixpyrrole chemistry have been concerned with their anion selective behaviour^41–44^ through the NH functionalities of the pyrrole rings in their structure, the possibilities offered by these macrocycles for modifying their structure either by replacing pyrrole units by thiophene or by introducing suitable pendent arms^45^ have led to a number of selective complexing agents for cations. A number of calixpyrrole based oligomers have been reported^46–48^ which have shown interesting properties as extracting agents mostly for anions.

**Fig. 1:**
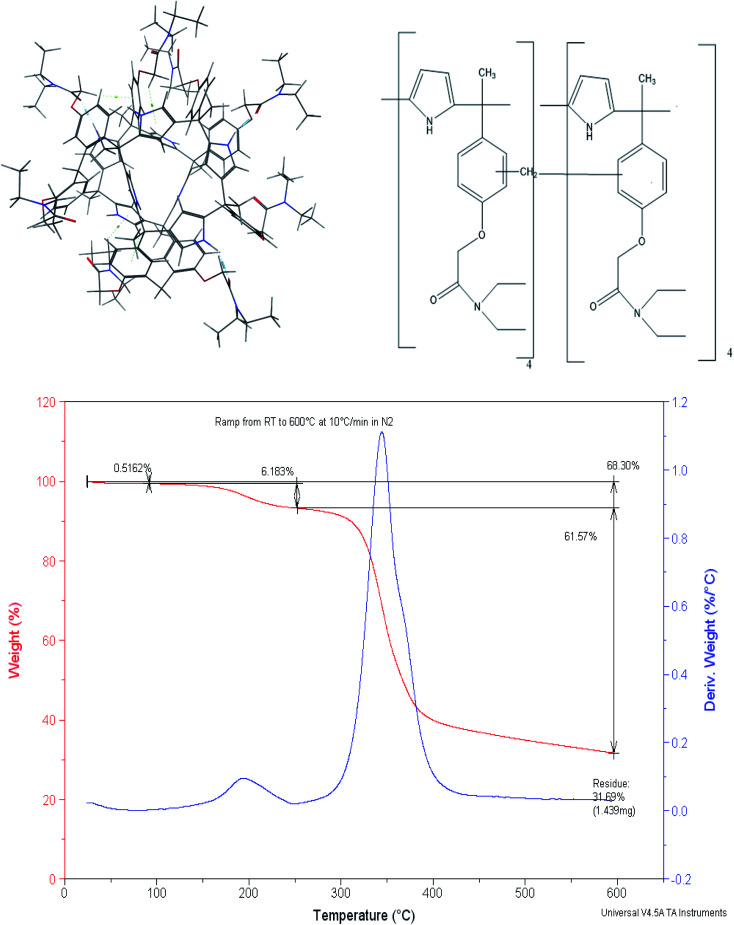
2D and 3D structure and TG curve of the CPA dimer.

We have previously reported a mercury(ii) ion selective electrode based on a calix[4]pyrrole amide derivative (*meso*-tetramethyl-tetrakis-(4-*N*,*N*-diethylacetamide phenoxymethyl)calix[4]pyrrole) (CPA),^49^(Fig. 1), and more recently demonstrated the role of thermodynamics in the selection of the receptor to be introduced in the ion selective membrane.^50^ This led us to explore the ability of the dimer based on this receptor to extract selectively mercury(ii) relative to other cations from water. In doing so the calix[4] pyrrole amide receptor was treated with formaldehyde in acid medium to obtain the dimer. Therefore the aim of this paper is to report (i) the synthesis and characterisation of the calix[4] pyrrole amide based new material and (ii) its extraction properties for the removal of mercury(ii) from water at a laboratory scale in single and multiple component metal ion solutions. (iii) Molecular simulation studies to assess the effect of the counter ion and on possible receptor-humic acid competition in real water systems.

## Experimental

### Chemicals

Metal cation salts (as nitrates) were purchased from Aldrich. These were dried and stored over phosphorus pentoxide, P_4_O_10_ in a vacuum desiccator at room temperature for several days prior to use, the absence of a water signal in the NMR spectra upon addition of metal cation salts pointed out that these salts were dry.

Solvents purchased from Fisher Scientific were refluxed in a nitrogen atmosphere followed by distillation and the middle fraction was collected.^51^ However, all solutions used in the ^1^H NMR measurements were prepared in deuterated solvents. Reagents used for the synthesis of the CPA receptor are those previously reported.^49^

### Dimerization of *meso*-tetramethyl-tetrakis-[(*N*,*N*-diethyl-acetamide)phenoxy-methyl] calix[4]pyrrole^52^

The synthesis of the CPA dimer was performed by condensation of *meso*-tetramethyl tetrakis [(*N*,*N*-diethylacetamide)phenoxymethyl] calix[4]pyrrole, CPA with formaldehyde in the presence of toluene, C_7_H_8_ as an inert continuous phase. The solution of CPA (2.0 g, 2.7 mmol), and 37% formaldehyde (3.33 cm^3^, 43 mmol) were dispersed in toluene (60 cm^3^) and stirred in a 100 cm^3^ reactor under reflux for 90 min at 90 °C. Thereafter, the CPA dimer was yielded as a solid, insoluble product in the form of beads which were collected, filtered-off, washed with ethanol, then water followed by acetonitrile.

### Dimer characterisation

The dimer was characterised by mass spectrometry (using a Bruker Autoflex Maldi-Tof Mass Spectrometer), thermogravimetric analyser (TGA Q500 V6.7), FT-IR spectrometry (using an Agilent Cary 600 Series FT-IR spectrometer with MKII Golden Gate Single Reflection ATR System). The infrared spectra were recorded by averaging 32 scans at a resolution of 4 cm^−1^ in the region of 500–4000 and scanning electron microscope (SEM) JEOL JSM-7100F, equipped with secondary and backscattered imaging coupled with ultra dry energy dispersive X-ray (EDX) for elemental analysis. For electron microscope imaging, samples were coated with a thin film of gold. Working conditions were 15 keV for accelerating voltage and 10 mm for the detector working distance.

### Molecular simulation and modelling

The structure of the synthesised material was modulated using MOE 2014.09 software. Steric energies of the ligand and the complexes were evaluated by Merck Molecular Force Field parameters (MMFF94x).

The strain energy of the complex was calculated by using a potential energy function, which contains terms for the strain in the bonds, angles, torsion angles, non-bonded interactions and charge interactions such as,*E*(*x*) = *E*_str_ + *E*_ang_ + *E*_stb_ + *E*_oop_ + *E*_tor_ + *E*_rdw_ + *E*_ele_ + *E*_sol_ + *E*_re_where:
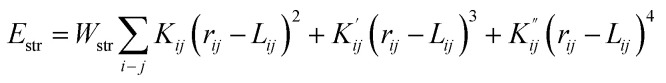
*etc.*

Hence any deviation from the expected bond lengths, angles, *etc.* leads to strain. Complexes tend to be stable if they do not have any strain energy, those that do are unstable. The molecular mechanics modelling shows in this case that the complex has strained angles and torsions and hence will prefer to relieve this strain by reacting.

### Extraction of cation salts from water by the CPA dimer

The ability of the CPA dimer to extract heavy metal ions (Hg(ii), Pb(ii), Zn(ii), Cu(ii) and Cd(ii)) (as nitrate salts) from aqueous solutions was investigated using the batch technique. The effect of various parameters such as amount of dimer, initial metal ion concentration, pH of the aqueous solution, kinetics and temperature were investigated with the aim of establishing the optimal conditions for the extraction process.

### Effect of the amount of ligand on the metal ion extraction from aqueous solution

The optimal amount of the CPA dimer for the extraction of metal ions from aqueous solutions was determined using different quantities of the dimer (0.02–0.2 g) and a known volume (10 cm^3^) and concentration (1 × 10^−3^ mol dm^−3^) of an aqueous solution of the metal ion salt at a pH of 5.6. Tubes were sealed and mechanically shaken for 5 min then left for 24 hours to equilibrate. Samples of aqueous solutions were taken and initial, *c*_i_ and equilibrium, *c*_e,_ metal ion concentrations were determined by atomic absorption spectrometry (AAS) or ICP/MS, except mercury ion which was analysed by potentiometry using a Hg(ii) ISE or ICP/MS. The extraction percentage (% *E*) was calculated using eqn (1)1
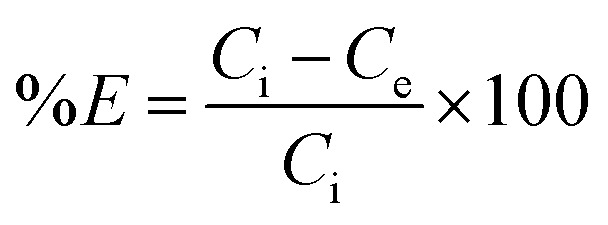


### The effect of solution pH on the extraction

An aqueous solution of each metal ion salt (10 cm^3^, 1.0 × 10^−3^ mol dm^−3^) was placed in a tube containing an optimum mass of the dimer. pH of samples were adjusted to desired values (in the 2–11 range) by adding a few drops of nitric acid, HNO_3_ or sodium hydroxide, NaOH solution [0.1–1.0 mol dm^−3^]. All samples were shaken for 5 min and then kept in a thermostat water bath at a fixed temperature (298 K) for 24 h. After this period, samples were taken and analysed as described above.

### Determination of the capacity of the material to extract cations

The capacity of the CPA dimer to take up metal cations (maximum amount of metal cations per gram of material) was investigated using batch experiments. Ten volumetric flasks containing a volume (10 cm^3^) of aqueous solutions of salts (concentration range of 2.0 × 10^−4^ to 2.0 × 10^−2^ mol dm^−3^) except for Hg(ii) (1.6 × 10^−6^ to 2.0 × 10^−2^ mol dm^−3^) were prepared by diluting appropriate amounts from stock solutions of the appropriate metal ion salt. The optimal mass of the CPA dimer was added to each test tube containing the solution. All samples were adjusted to optimal pH (pH = 5.6). Contents of test tubes were shaken for about 5 min and then kept overnight in a thermostat water bath at a fixed temperature (298 K). After equilibration, initial and equilibrium concentrations were determined as described above. The capacity of the material to remove cations *Q*_e_, was calculated from eqn (2)2
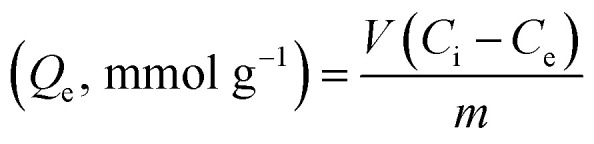
where *V* is the volume (dm^3^) of the sample solution and *m* is the mass (g) of the extracting agent used.

The capacity of the dimer in the presence of mercury(ii) and other cations (Pb(ii), Zn(ii), Cd(ii) and Cu(ii) salts ∼1.0 × 10^−2^ mol dm^−3^) was determined as described above.

### Kinetics of the extraction process

The effect of contact time on removing metal ions from aqueous solutions by the dimer was investigated at different time intervals (5, 20, 40, 60, 80, 100 and 120 minutes). An optimum amount of the CPA dimer was added to seven tubes (50 cm^3^). A fixed volume (10 cm^3^) of the optimal concentration and pH was added to each tube. These were placed in a thermostat water bath at constant temperature (298 K). After different periods, samples were removed from the water bath. The metal ion concentration in each tube was determined as described above.

### Effect of temperature on the extraction process by the CPA dimer

The effect of temperature on removing metal ion salts from aqueous solutions by the dimer was examined within the 298–343 K range while all other parameters were kept constant. Thus, a series of aqueous metal ion salt solutions (10 cm^3^) were left in contact with the dimer overnight in a water bath at different temperatures (298, 303, 313, 323, 333 and 343 K). Aliquots of the aqueous solution were taken and analysed.

## Results and discussion

### Characterization of the CPA dimer

The presence of phenyl groups in the structure of the CPA monomer led us to explore its treatment with formaldehyde to produce the dimer in which the two monomers are joined by a methylene bridge. It is essentially based on the phenol–formaldehyde reaction^52–54^ which was later on used to produce resins containing crown ethers as anchor groups^55–57^ and more recently calix[4] based dimers^46,58^ reported by Danil de Namor and co-workers. Molecular Simulation of the CPA dimer (Fig. 1) shows two monomers joined by a –CH_2_– linkage. The dimer characterised by mass spectroscopy (MS) (ESI, S1†) shows a residual peak at 2420.3 g mol^−1^. Given that the molecular weight of the calix[4]pyrrole derivative, CPA, is 1193.68 g mol^−1^ and considering the –CH_2_– linkage and the presence of a water molecule shown in the molecular simulation it follows that the suggested model is in agreement with the experimental value obtained from MS. The dimer was also characterised by thermal analysis (TGA-DTA) and scanning electron microscope (SEM) equipped with energy dispersive X-ray (EDX).

As far as the thermogravimetric analysis is concerned, the thermogram (Fig. 1) shows that the dimer can preserve thermal stability up to 280 °C. Two exothermic peaks are shown in the TG curve. The first one which corresponds to the release of moisture from the surface (average experimental mass loss of 6.18%) is followed by the second one which corresponds to the decomposition of the material with an average mass loss of 61.57%.

**Fig. 2:**
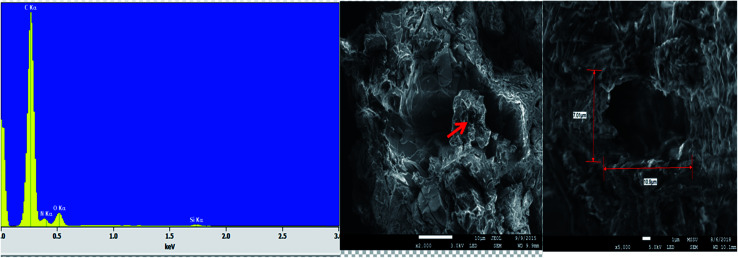
SEM-EDX surface analysis of CPA dimer.

The energy dispersive X-ray analysis (EDX) of CPA dimer displays the characteristic peaks that reveal the elemental composition (C, N, and O). The dimer morphology illustrated by SEM (Fig. 2) shows an uneven aggregate, rough and porous surface. From the given dimensions in Fig. 2, the dimer appears to be microporous.

### Analysis of unloaded CPA dimer and loaded CPA with metal cations

**Fig. 3:**
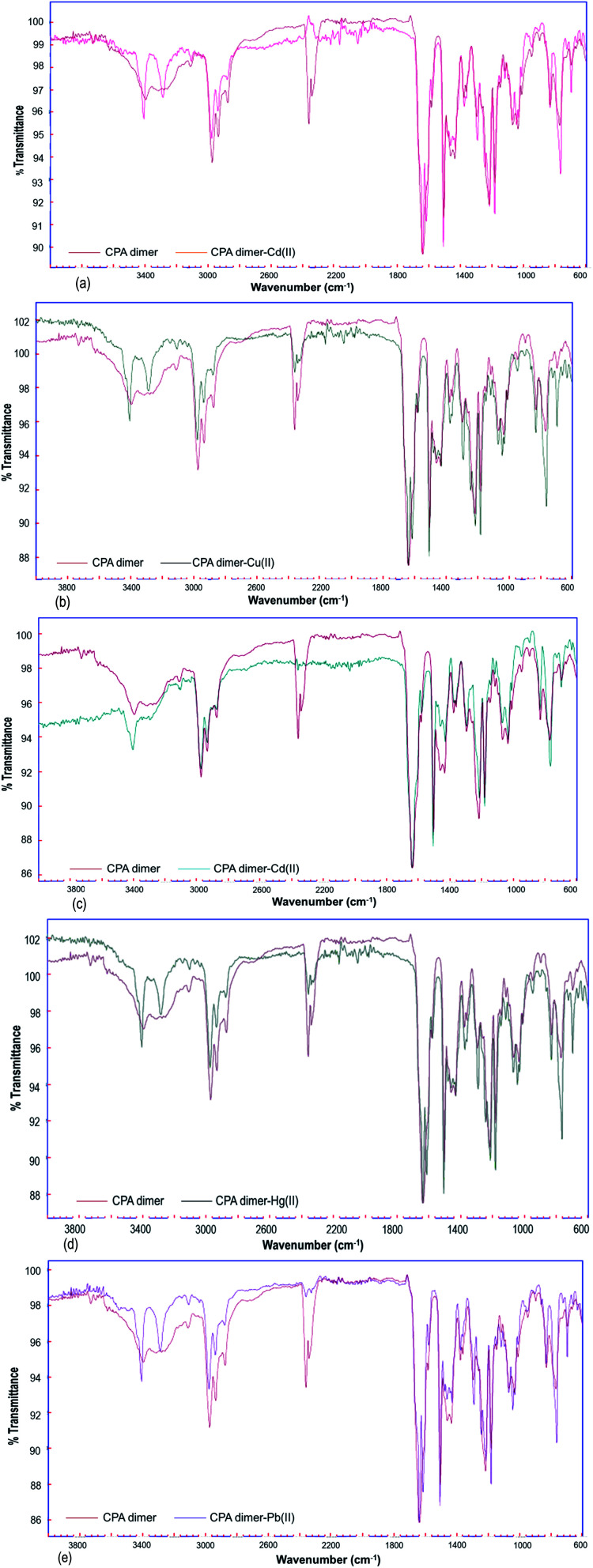
FTIR spectra of unloaded and loaded CPA dimer with Zn(ii) (a), Cu(ii) (b), Cd(ii) (c), Hg(ii) (d), Pb(ii) (e).

Fourier transform infrared (FTIR) spectra were obtained to identify the characteristic functional groups of the dimer that could be responsible for the extraction of heavy metal ions from aqueous solution. All FT-IR spectra (Fig. 3) either for unloaded or loaded dimer were measured within the range of 4000 to 600 cm^−1^.

The FT-IR spectrum of the native CPA dimer sample (unloaded sample) showed the presence of different functional groups. The absorption peak at 3394 cm^−1^ indicates the presence of hydration. The peak observed at 3312 cm^−1^ can be assigned to NH groups of the pyrrole ring.

The band located in a region of 2990 is due to the presence of the C–H aromatic while the peak observed at 2935 represents the C–H aliphatic (CH_3_ group). The CH_2_ bond was observed at 2950 cm^−1^. So, the signal peaks located at 2340 and 2360 cm^−1^ are due to the existence of stretching modes associated with the presence of occluded CO_2_ in the material pores caused by thermal treatment of the sample during the dryness process. The peak at 1639 cm^−1^ represents the C=O group (amide bond). Prominent peaks were at 1615, 1581, 1462 and 1435 cm^−1^ arising from the presence of C=C aromatic (pyrrole and benzene). The bonds observed in the range 1219–1182 cm^−1^ represent ether C–O–C bond.

On the other hand, FT-IR spectra of the metal cation-loaded dimer showed small shifts in the absorbance of some peaks attributed to the (–NH) functionality as compared with the spectrum of the unloaded CPA. The band at 3312 cm^−1^ (–NH) was shifted to 3287 when the dimer was loaded with Hg(ii) or Pb(ii) ions. This band (–NH) located at 3312 cm^−1^ was also shifted to 3284, 3285 and 3294 cm^−1^ caused by loading the dimer with Zn(ii), Cu(ii), and Cd(ii) cations respectively.

It can therefore be concluded from the FT-IR spectral results for the unloaded and loaded CPA dimer bands assigned to C–H aliphatic, C=C aromatic, CH_2_, ether C–O–C groups did change but not significantly. This reveals that these functional groups are not responsible for binding the dimer with metal cations. Thus, the interaction of the CPA dimer with metal cations seems to occur through the carbonyl oxygens of the acetamide functional groups.

### SEM-EDX analyses of unloaded and loaded CPA dimer with Zn(ii), Cu(ii), Cd(ii), Hg(ii) and Pb(ii)

**Fig. 4:**
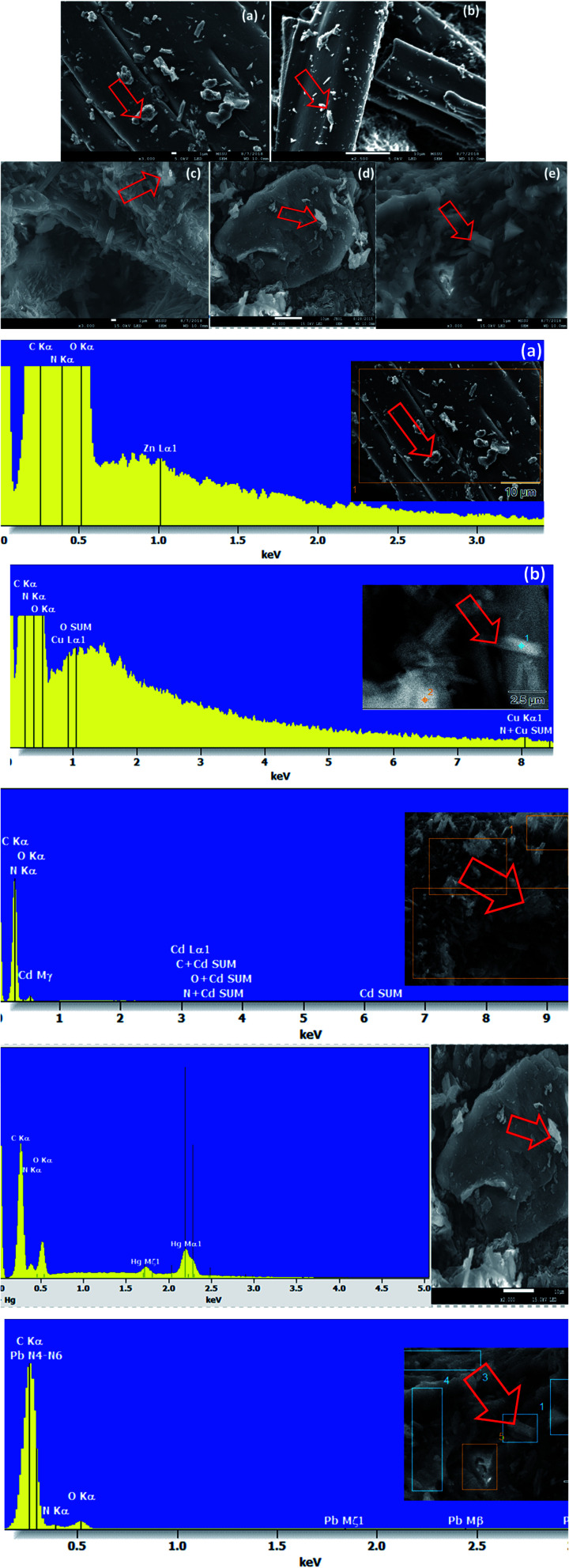
Scanning electron micrographs and EDX spectra showing the elemental composition of CPA dimer treated with Zn(ii) (a), Cu(ii) (b), Cd(ii) (c), Hg(ii) (d) and Pb(ii) (e). Al peak referred to the aluminium stub used for samples' mounting.

SEM and X-ray (EDX) studies undertaken prior and after equilibration with Zn(ii), Cu(ii), Cd(ii), Hg(ii) and Pb(ii) aqueous solutions provide a clear evidence of the presence of these ions on the surface of the dimer (Fig. 4). The micrographs display additional small white patches (arrow) which are formed due to the complexation with the dimer which are not shown in the micrograph of the unloaded dimer. Another striking feature of the micrographs of the dimer loaded with the investigated metal cations (Fig. 4) is the change in their morphology with the cation. Thus the CPA dimer complexed with Zn(ii) and Cu(ii) appear to have a cylindrical shape (a) and (b) while aggregate shapes are observed with the dimer loaded with Cd(ii) (c) and Pb(ii) and (e). The dimer loaded with Hg(ii) (d) looks as large clump with smooth surface. The introduction of metal ions in the structure of the dimer provides an external stimulus with considerable changes in morphology due to conductivity changes which will be dependant of the metal cation salt and consequently will lead to changes in their physical properties.^59^

The EDX spectra for the metal ion loaded dimer (Fig. 4) shows different peaks for these metals ions which clearly indicate that these ions have been uptaken by the dimer which confirm the specific presence of Zn(ii), Cu(ii), Cd(ii), Hg(ii) and Pb(ii) ions onto the surface of the material. These findings do not exclude the possibility of interactions not only on the surface but also inside the dimer.

Having characterised the free and loaded dimer, the extracting properties of the dimer were assessed.

### Extraction of metal cations from aqueous media by the CPA dimer under different experimental conditions

The optimal conditions for removing metal cations by the calix[4] pyrrole based dimer were investigated taking into account the mass of material, pH of the aqueous solution, temperature, the kinetics of the process and the removal capacity of the material for metal cations in a single and multiple component meta-ion solutions as well as its recycling.

### Effect of the amount of the CPA dimer on the extraction process

**Fig. 5:**
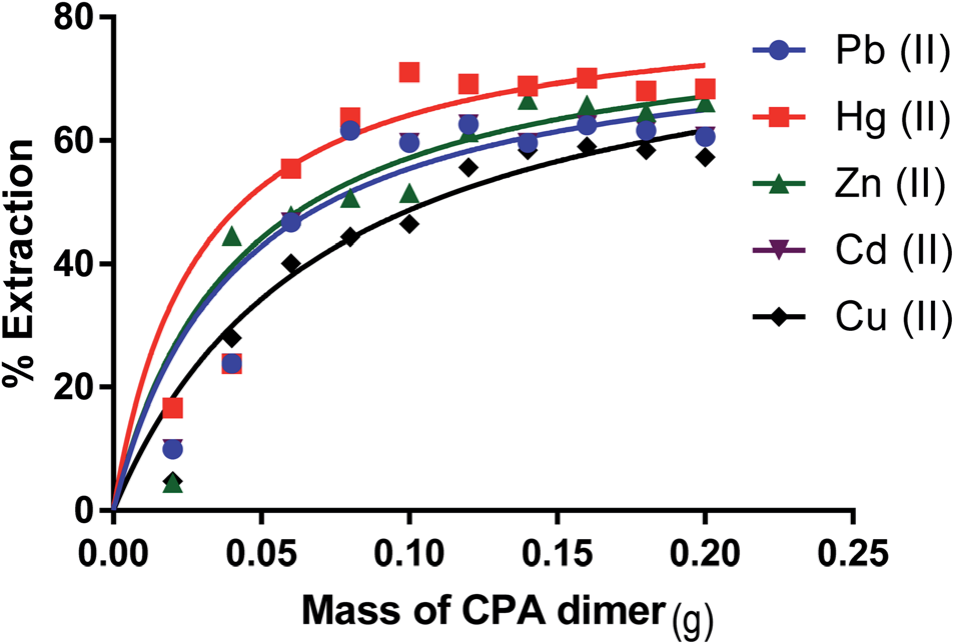
Effect of CPA dimer mass on the extraction of Zn(ii), Cu(ii), Cd(ii), Hg(ii) and Pb(ii).

Removal percentages (%) (eqn (1)) for Zn(ii), Cu(ii), Cd(ii), Hg(ii) and Pb(ii) from aqueous solution as a function of the amount of the dimer are shown in Fig. 5 respectively. It is observed that the removal percentage of cations increases gradually with raising the amount of CPA. This is attributed to an increase in the binding sites of the dimer. On the other hand, further addition of the dimer did not cause any significant change in the removal% of these salts from aqueous solutions. This indicates that the maximum removal percentage is attained when an amount of the CPA dimer is ∼0.1 g.

### Effect of the solution pH on the removal of metal ion salts by the CPA dimer

**Fig. 6:**
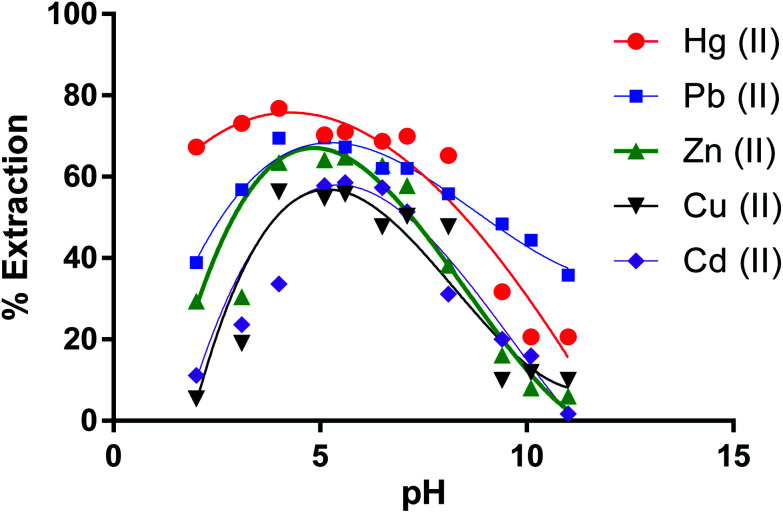
Effect of pH on the extraction percentage of Zn(ii), Cu(ii), Cd(ii), Hg(ii) and Pb(ii) by CPA dimer.

Removal percentages of cations by the dimer *versus* pH values (Fig. 6) show that the % *E* by the dimer for most cations at the lower and higher pH was low At pH lower than 4.0 (except for Hg(ii)), H^+^ ions may compete with metal ions on the extraction sites while at pH higher than 7, metal ions start to precipitate as metal(ii) hydroxides [M(OH)_2_] due to increasing concentration of OH^−^ ions in solution.^60^

From these results, it can therefore be concluded that the extraction efficiency (%) of the dimer for metal ions at optimum pH values increases according to the following sequence,Hg(ii) > Pb(ii) > Zn(ii) > Cd(ii)> Cu(ii)

Therefore, the solution pH value for the experimental investigation of the extraction process for further work was set at the optimum value obtained.

### Capacity of the dimer to extract cations from water

**Fig. 7:**
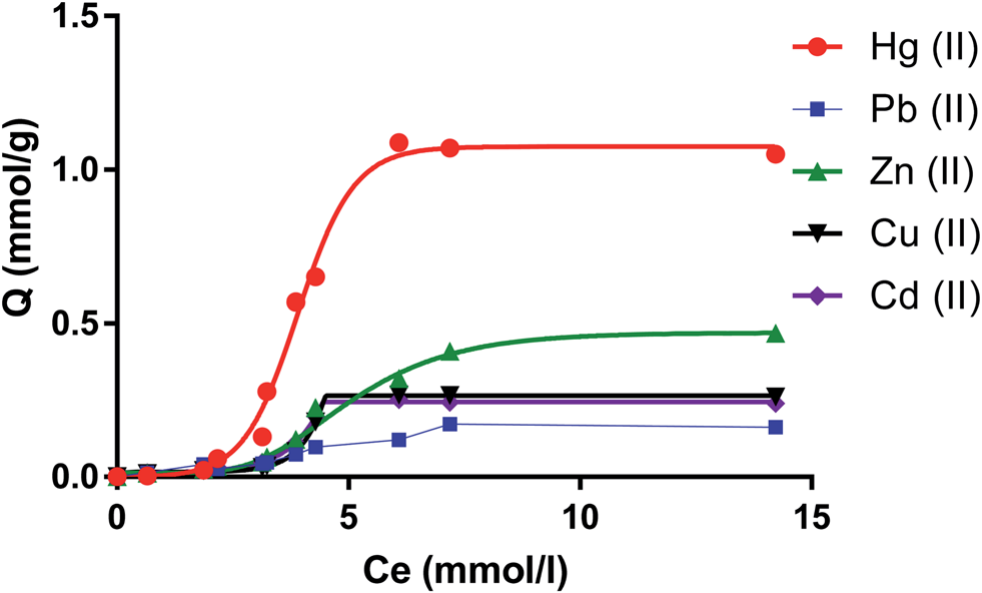
Uptake capacity (mmol g^−1^) of CPA dimer of Zn(ii), Cu(ii), Cd(ii), Hg(ii) and Pb(ii).

The amount of metal ions on the molar scale per gram of dimer against the molar concentrations of salts is shown in Fig. 7. Numerical values for the capacity of the dimer to uptake metal cations in mmol as well as in mg (between brackets) per gram of the dimer are shown in Table 1.

**Table 1:** Capacity of a dimer for one and multiple component metal solution. Selectivity factor (*S*) Hg(ii) relative to other cations^a^

Cation	*Q*_e_ one component (mmol g^−1^ or mg g^−1^)	*S*	*Q*_e_ multiple component (mmol g^−1^ or mg g^−1^)	*S*
Hg(ii)	1.07 or 214.63	1	0.48 or 96.28	1
Zn(ii)	0.46 or 30.07	2.5	0.01 or 0.65	48
Cu(ii)	0.26 or 16.52	4.1	0.03 or 1.9	16
Cd(ii)	0.24 or 26.97	4.4	0.01 or 1.12	48
Pb(ii)	0.16 or 33.15	6.7	0.12 or 24.86	4

a *Q*_e_ is the maximum amount of metal cations per gram of material in mmol g^−1^ or mg g^−1^. Multiple component medium contain equimolar concentration of Hg(ii), Pb(ii), Zn(ii), Cu(ii) and Cd(ii) (as nitrate salts).

The selectivity of the monomer for mercury has been quantitatively reported from complexation thermodynamic data involving metal cations.^49^ This is also reflected in the ability of this dimer to selectively extract Hg(ii) relative to remaining cations. In fact from data expressed on the molar scale it follows that the uptake of Hg(ii) by this receptor is higher by factors of over 2, four and six relative to Zn(ii), Cu(ii) and Cd(ii) (∼the same) and Pb(ii) respectively. These results refer to dimer's capacities towards one component metal-ion solution. There are cases in wastewater treatments that knowledge regarding the dimer's capacities in multi-component solutions containing all these cations are required. The results also reported in Table 1 are striking in that the capacity values have been altered significantly to the extent that the presence of Cu(ii), Zn(ii) and Cd(ii) will hardly interfere on the removal of Hg(ii) by the dimer from water. This is not the case for Pb(ii). The capacity of the dimer for this cation in a multi-component solution is not altered significantly. Consequently the capacity of the dimer for Hg(ii) has decreased. However the selectivity of the dimer for Hg(ii) still remains. Calculation of the selectivity factors (*S* = *Q*_e_(Hg(ii)/*Q*_e_M(ii))) (Table 1) shows a dramatic increase in the *S* values for Hg(ii) relative to other cations while a decrease is found in the *S* value for Hg(ii) relative to Pb(ii). The fact that the dimer although selective for Hg(ii) is able to extract Pb(ii) is an important finding given that this cation is also potentially toxic and it is often found in water due to anthropogenic activities.^61^ An explanation of these results relies on the Gibbs energies of hydration of these cations, Δ_hyd_*G*°, (the contribution of the counter-ion also present will be the same as all salts used have a common counter-ion) given that in the process of cation transfer from water to the neutral dimer, dehydration will take place. In a multi-component system the kinetic factor will play a predominant role leading to competition. Thus Pb(ii) seems to compete successfully with Cu(ii), Zn(ii) and Cd(ii) and to a certain extent with Hg(ii). This must be attributed to the lower Δ_hyd_*G*° of Pb(ii) = (−1425 kJ mol^−1^) relative to Cu(ii) (−2010 kJ mol^−1^), Zn(ii) (−1955 kJ mol^−1^), Cd (−1755 kJ mol^−1^) and Hg (−1760 kJ mol^−1^). The fact that the dimer shows selectivity for Hg(ii) relative to Pb(ii) must be attributed to the higher hosting ability of the monomers components of the dimer for Hg(ii) (2 cations per unit of receptor) relative to any other cation as previously demonstrated.^49^

### Effect of contact time on the extraction of cations by CPA dimer

**Fig. 8:**
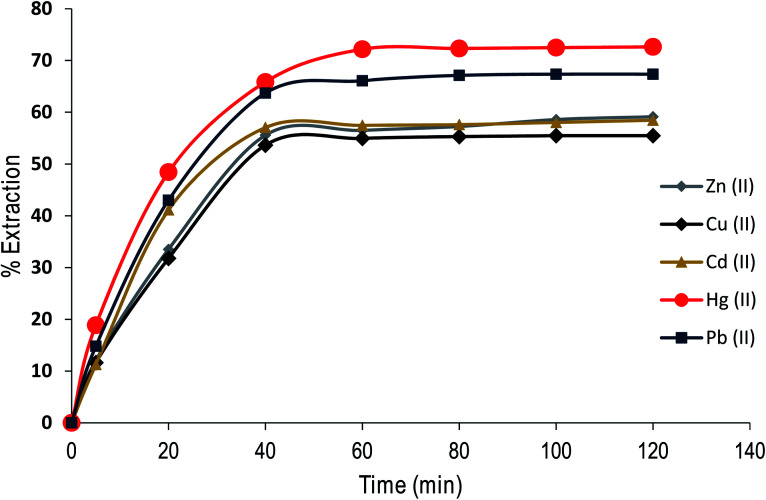
Effect of contact time on Zn(ii), Cu(ii), Cd(ii), Hg(ii) and Pb(ii) extraction from aqueous solution by CPA dimer at 298 K.

The contact time is an important parameter in extraction studies. Fig. 8 shows the results obtained in assessing the influence of contact time on the % *E* of metal cations (as nitrate salts) from aqueous solution under optimal experimental conditions using the dimer.

The experimental data were used to calculate kinetic parameters by using a simple exponential model as the rate of the uptake of the dimer is proportional to the distance from equilibrium (*i.e.* pseudo first-order process). By fitting the data points, the half-lives *t*_1/2_ and rate constants (*k*) are as follows, Zn(ii) *t*_1/2_: 14 min; *k* = : 0.047, Cu(ii) *t*_1/2_: 14 min; *k* = 0.047, Cd(ii): *t*_1/2_: 12 min; *k* = 0.058, Hg(ii) *t*_1/2_: 12 min; *k* = 0.056, Pb(ii) *t*_1/2_ 13 min; *k* = 0.053. These results demonstrate that the removal process is fast, an important issue to consider for commercialisation purposes.

### Effect of the counter-ion on the metal ion extraction processes

The experimental work on the extraction of cations from water by the dimer was carried out using nitrate as the counter-ion. Molecular simulation studies (see Table 2). Fig. S2 given in the ESI† shows simulation studies on the effect of each counter-ion on the interaction with the dimer. The outcome reflects that as far as the counter-anions are concerned the stability follows the sequence,ClO_4_^−^ > Cl^−^ > NO_3_^−^which means that the extraction of these cations would be greater when perchlorate or chloride rather than nitrate is the counter-ion. A direct consequence of this statement is that in the presence of chloride, the most abundantly found anion in water the capacity to uptake cations and mainly Hg(ii) would be even greater than the one reported here. Given that the active functional groups of the dimer are amides, as a matter of interest it is relevant to consider the single-ion transfer Gibbs energies, Δ_*t*_*G*° (data based on the Ph_4_As Ph_4_B convention)^62^ of these anions from water to a solvent containing functional groups similar to those in the dimer such as *N*,*N*-dimethylformamide (DMF). In fact the trend observed in these data is the same as that obtained from molecular simulation calculations as far as ClO_4_^−^ and Cl^−^ anions are concerned. Thus Δ_*t*_*G*° values of perchlorate and chloride from water to DMF are 5.06 and 48.6 kJ mol^−1^ respectively.

**Table 2:** Energy calculations on the effect of the counter-ion on the extraction of Hg(ii) from water by the dimer

Salt	Energy (kcal mol^−1^)
Hg(ClO_4_)_2_	−19675.8
HgCl_2_	−19625.9
Hg(NO_3_)_2_	−19369.3

### Competition between the dimer and humic acid for Hg(ii)

Being humic acid one of the main components of soil, its presence in real water samples is expected.

**Fig. 9:**
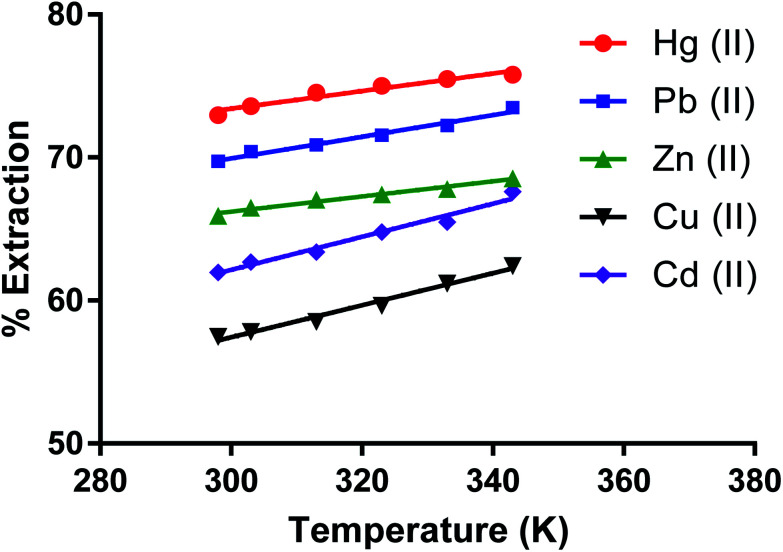
MD simulation in water, the humic acid and mercury(ii) have dissociated completely. Energy = −34.279 kcal mol^−1^ (AMBER 10: EHT force field).

Due to its wide variety of functional groups it is known to interact with metal cations. Therefore we proceeded simulation studies in an attempt to assess whether or not this material is able to compete with the dimer for the mercury(ii) cation. Thus NVT molecular dynamics simulations were run for 600 ps each in a periodic box of water (Fig. 9). The simulations contained in turn one, two and three humic acids complexed to mercury and 2091 water molecules. After running the simulations at room temperature the mercury completely dissociated from the humic acid after 30 ps in all of the simulations. In a competitive simulation with the mercury complexed to the CPA dimer and also containing 3 humic acids, the mercury dissociates from the CPA dimer after 100 ps and does not spend any time complexed to any of the humic acids indicating that the complexation with the dimer is stronger than that with the humic acids and also that the humic acids are not able to remove the mercury from the CPA dimer in these simulations. Given that the functional groups which are responsible for the reactivity of humic acid and contribute to its surface charge are thought to be phenolic and carboxylic functionalities^63^ these have a higher affinity for hard metal cations such as Na(i), (Mg(ii) rather than soft ones like Hg(ii))^64^ the outcome of simulation studies seem to be reasonable.

### Effect of temperature on removing metal ions by the dimer

**Fig. 10:**
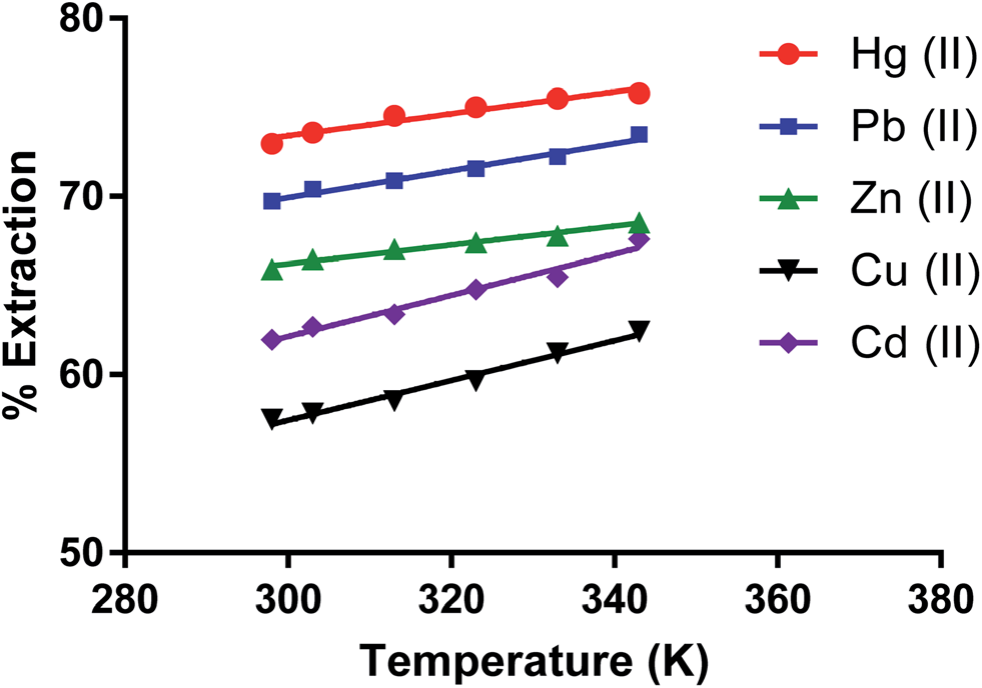
Effect of temperature on the extraction of Zn(ii), Cu(ii), Cd(ii), Hg(ii), Pb(ii) salt by the CPA dimer.

The effect of temperature (*K*) on removing metal ion salts (as nitrates) from an aqueous solution by CPA under optimum conditions (Fig. 10) in the 298 to 343 K range shows relatively small changes in the % *E* with temperature. This is expected given the several individual processes taking place in the overall extraction of cations by the dimer as previously discussed for solvent extraction processes^65,66^ involving univalent ions. The complexity of the system is greater when bivalent cations are involved. The derivation of the enthalpy change by the van't Hoff equation may lead to misleading values due to its limitations. The main one is that the heat capacity of the system is not considered.^67^ Even though, for the derivation of enthalpy data from this equation, accurate equilibrium constants are required.^68^ There have been interesting papers regarding the determination of thermodynamic parameters for adsorption processes.^68–73^ However when macrocycles are involved a rigorous thermodynamic approach cannot be applied for the following reasons.

(I) Although there are indications from SEM-EDX that some cation-dimer complexation takes place at the surface, there is no reason to consider that the cation cannot penetrate the surface of the dimer. Therefore the process cannot be regarded as an adsorption process.

(II) It is often found from calorimetry that heats associated with these processes are relatively small to the point that cannot be accurately measured, due to the number of individual processes involved such as transfer of the ionic salt from water to the neutral material, de-solvation of the cation upon complexation, greater for bivalent than univalent cations (endothermic process), cation-dimer complexation (exothermic process) ion pair formation between complex cation and anion (slightly exothermic) due to the fact that the proximity of complexed cation and anion significantly decreases given that the size of the complexed cation is larger than the free cation to the extent that some slight conductivity of the solid material loaded with the salt may be observed. All these facts add complexity in the definition of a standard state for the solid phase. Under these conditions it is pointless to derive thermodynamic data for undefined processes.

### Recycling the dimer

**Fig. 11:**
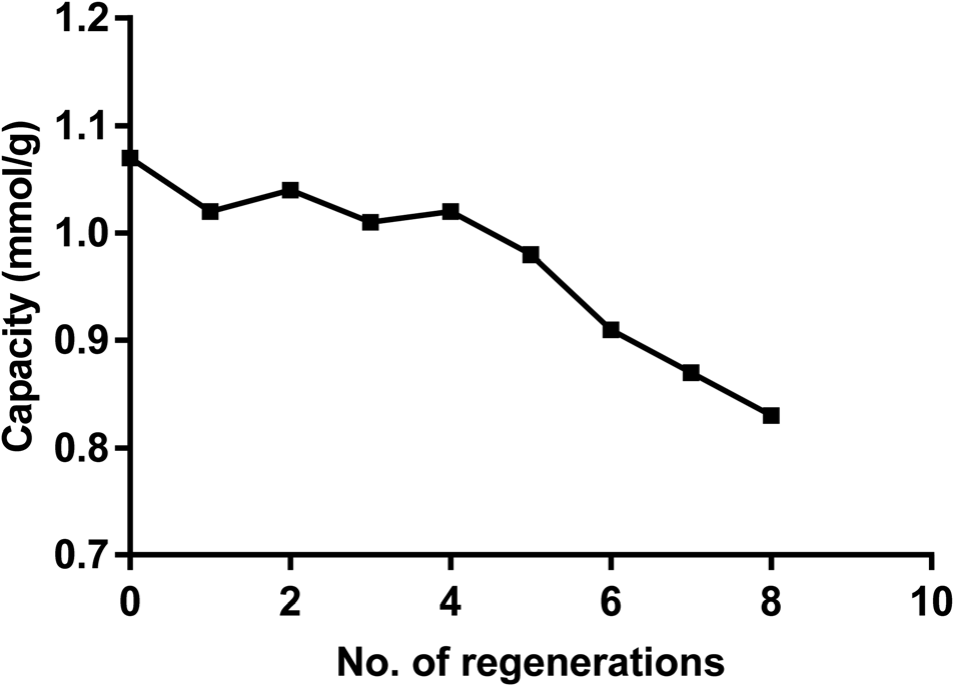
Capacity of the dimer for Hg(ii) after several recycling.

From data reported in Fig. 6 it follows that the dimer can be easily recycled *via* a pH switching mechanism either at very low pH using HNO_3_ 1 M or at high pH by treatment of NaOH. As for other calix[4] based receptors and materials the recycling of the dimer was carried out by a pH switching mechanism as previously reported.^74,75^Fig. 11 shows that after several recycling processes the loss in the removal capacity for Hg(ii) was not greater than 20%.

### Comparison with other materials

In attempting to establish comparisons between the selectivity of the dimer for mercury with previously reported work^76–86^ in this area it is found that the concept of selectivity has not been carefully considered. A considerable amount of work has been carried out on materials able to take up mercury from aqueous medium but some of these studies should be revisited due than in some cases interferences by other cations have not been assessed^77–80,82^ which means that the selectivity issue has not been addressed. The anion effect has not been considered and therefore it is not possible to determine the selective behaviour of the material for mercury given that salts used have different counter-ions,^82^ the removal capacity of the material is greatly reduced to 74 and 58% after a single recycling process limiting their commercial applications of these materials,^74^ the temperature at which these studies have been carried out is either not reported or referred to an undefined room temperature.^74,77,79,82^ Capacity values derived from Langmuir^76,83,84^ or Redlich–Peterson^81^ equations rather than experimental values were reported. Thiol containing polymer encapsulated magnetic nanoparticles^86^ as well as amino functionalised mesoporous silica^78^ have been tested but they are not selective for mercury(ii).

## Conclusions

From the above discussion the following conclusions are drawn

(i) A new dimer based on a calix[4]pyrrole amide derivative selective for Hg(ii) in one and in multiple component solutions was synthesised and characterised.

(ii) FTIR, SEM and EDX measurements showed that the dimer interacts with Zn(ii), Cu(ii), Cd(ii), Hg(ii) and Pb(ii). The presence of these ions on the surface of the dimer was identified by SEM and EDX but this finding does not exclude the possibility of the cation penetrating the surface of the dimer.

(iii) Optimal conditions for removing cations from water were established.

(iv) Single-ion Gibbs energies of transfer (based on the Ph_4_AsPh_4_B) from water to a solvent containing a common functionality to that of the material were used to assess the counter-ion effect on the removal process. Agreement with results obtained from molecular simulation and preliminary studies were obtained. The use of transfer data to avoid unnecessary experimental work is emphasised.

(v) Molecular simulation studies on water components (humic acid) and the dimer in the presence of mercury(ii) show that humic acid is not able to compete with the dimer for this cation.

Finally, based on the fundamental research reported here the use of this material at pilot plant scale is in progress. In doing so BET measurements, size of the material as well as testing the material in real systems involving contaminated waters in the mining areas of Argentina will be carried out.

## Conflicts of interest

There are no conflicts of interest to declare

## Supplementary Material

RA-010-C9RA09911E-s001

## References

[cit1] Bernhoft R. A. (2012). J. Environ. Public Health.

[cit2] Fernandes Azevedo B., Barros Furieri L., Peçanha F. M., Wiggers G. A., Frizera Vassallo P., Ronacher Simões M., Fiorim J., de Batista P. R., Fioresi M., Rossoni L., Stefanon I., Alonso M. J., Salaices M., Vassallo D. V. (2012). BioMed Res. Int..

[cit3] Gibb H., Leary K. G. (2014). Environ. Health Perspect..

[cit4] Grandjean P., Satoh H., Murata K., Eto K. (2010). Environ. Health Perspect..

[cit5] Houston M. (2011). J. Clin. Hypertens..

[cit6] Li R., Wu H., Ding J., Fu W., Gan L., Li Y. (2017). Sci. Rep..

[cit7] Yusà V., Pérez R., Suelves T., Corpas-Burgos F., Gormáz M., Dualde P., Coscolla C., Quiles J., Roca M., Vento M. (2017). Chemosphere.

[cit8] Science for Environment Policy, Tackling Mercury Pollution in the EU and Worldwide, In-depth Report 15 produced for the European Commission, DG Environment by the Science Communication Unit, UWE, Bristol, 2017, http://ec.europa.eu/science-environment-policy

[cit9] Åkerblom S., Bignert A., Meili M., Sonesten L., Sundbom M. (2014). Ambio.

[cit10] Brambilla G., Abete M. C., Binato G., Chiaravalle E., Cossu M., Dellatte E., Miniero R., Orletti R., Piras P., Roncarati A., Ubaldi A., Chessa G. (2013). Regul. Toxicol. Pharmacol..

[cit11] Da Cunha L. R., da Costa T. H. M., Caldas E. D. (2013). Biol. Trace Elem. Res..

[cit12] Diéguez M. C., Garcia P. E., Bencardino M., Amore F. D., Castagna J., Ribeiro Guevara S., Sprovieri F. (2016). Atmos. Chem. Phys. Discuss..

[cit13] Eagles-Smith C. A., Wiener J. G., Eckley C. S., Willacker J. J., Evers D. C., Marvin-Di Pasquale M. C., Obrist D., Fleck J. A., Aiken G. R., Lepak J. M., Jackson A. K., Stewart A. R., Webster J., Davis J. A., Alpers C. N., Ackerman J. T. (2016). Sci. Total Environ..

[cit14] Hacon S., Barrocas P. R. G., de Vasconcellos A. C. S., Barcellos C., Wasserman J. C., Campos R. C., Ribeiro C., Azevedo-Carloni F. B. (2008). Cad. Saúde Pública.

[cit15] Jürgens M., Johnson A., Jones K., Hughes D., Lawlor A. (2013). Sci. Total Environ..

[cit16] Kuballa T., Moellers M., Schoeberl K., Lachenmeier D. W. (2011). Eur. Food Res. Technol..

[cit17] Zhang L., Wong M. H. (2017). Environ. Int..

[cit18] Spiegel S., Keane S., Metcalfe S., Veiga M., Yassi A. (2014). Environ. Health Perspect..

[cit19] Gunatilake S. K. (2015). J. Multidisc. Eng. Sci. Stud..

[cit20] Dzionek A., Wojcieszyńska D., Guzik U. (2016). Electron. J. Biotechnol..

[cit21] Garbisu C., Alkorta I. (2003). Eur. J. Miner. Process. Environ. Prot..

[cit22] Mosa K. A., Saadoun I., Kumar K., Helmy M., Dhankher O. P. (2016). Front. Plant Sci..

[cit23] Sinha A., Khare S. K. (2012). Biodegradation.

[cit24] Bioremediation of Mercury: Current Research and Industrial Applications, ed. I. Wagner-Döbler, Caister Academic Press, USA, 2013

[cit25] Velásquez-Riaño M., Benavides-Otaya H. D. (2016). Crit. Rev. Biotechnol..

[cit26] Cobbett C. S. (2000). Plant Physiol..

[cit27] Bernard C., Roosens N., Czernic P., Lebrun M., Verbruggen N. (2004). FEBS Lett..

[cit28] Williams L. E., Pittman J. K., Hall J. L. (2000). Biochim. Biophys. Acta.

[cit29] Guerinot M. L. (2000). Biochim. Biophys. Acta.

[cit30] Hall J. L., Williams L. E. (2003). J. Exp. Bot..

[cit31] Paulsen I. T., Saier Jr M. H. (1997). J. Membr. Biol..

[cit32] Rauser W. E. (1999). Cell Biochem. Biophys..

[cit33] Idris S., Harvey S. R., Gibson L. T. (2011). J. Hazard Mater..

[cit34] Fernandes S., Eichenseer C. M., Kreitmeier P., Rewitzer J., Zlateski V., Grass R. N., Stark W. J., Reiser O. (2015). RSC Adv..

[cit35] Ganzagh M. A. A., Yousefpour M., Taherian Z. (2016). J. Chem. Biol..

[cit36] Khayyat Sarkar Z. V. (2018). Int. J. Nanosci. Nanotechnol..

[cit37] Nasrollahpour A., Moradi S. M. J., Moradi S. E. (2017). J. Serb. Chem. Soc..

[cit38] Moghimi A. (2007). Chin. J. Chem..

[cit39] Lehn J. M. (2017). Chem. Soc. Rev..

[cit40] Wang H., Feng Z., Xu B. (2017). Chem. Soc. Rev..

[cit41] Danil de Namor A. F., Shehab M. (2005). J. Phys. Chem. B.

[cit42] Danil de Namor A. F., Khalife R. (2010). Phys. Chem. Chem. Phys..

[cit43] Gale P. A., Sessler J. L., Kral V., Lynch V. (1996). J. Am. Chem. Soc..

[cit44] Sessler J. L. , Gale P. A. and Cho W.-S., in Anion Receptor Chemistry, ed. J. F. Stoddart, Royal Society of Chemistry, London, 2007, pp. 1–66

[cit45] Danil de Namor A. F., Abbas I., Hammud H. (2007). J. Phys. Chem. B.

[cit46] Danil de Namor A. F., Abbas I. (2010). Anal. Methods.

[cit47] He Q., Kelliher M., Bähring S., Lynch V. M., Sessler J. L. (2012). J. Am. Chem. Soc..

[cit48] Saha I., Lee J. H., Huang H., Kim T. S., Lee C. H. (2015). J. Chem. Soc., Chem. Commun..

[cit49] Danil de Namor A. F., El Gamouz A., Alharthi S., Al Hakawati N., Varcoe J. (2015). J. Mater. Chem. A.

[cit50] Danil de Namor A. F., Alharthi S., El Gamouz A., Al Hakawati N., Cox B. G. (2018). Electrochim. Acta.

[cit51] Furniss B. S. , Hannford A. J., Smith P. W. G. and Tatchell A. R., Vogel's Textbook of Practical Organic Chemistry, Longman Scientific and Technical, London, 5th edn, 1989

[cit52] Baekeland L. H. , *US Pat.*, 942699A, 1907

[cit53] Lu K. T., Luo K. M., Lin S. H., Su S. H., Hu K. H. (2004). Process Saf. Environ. Prot..

[cit54] Poljanšek I., Krajnc M. (2005). Acta Chim. Slov..

[cit55] Blasius E., Janzen K. P., Adrian W., Klein W., Klotz H., Luxemburger H., Mernke E., NguyEn V. B., Nguyen-Tien T., Rausch R., Stockemer J., Toussaint A. (1980). Talanta.

[cit56] Danil de Namor A. F., Sigstad E. (1986). Polyhedron.

[cit57] Sigstad E. E. , PhD Thesis, University of Surrey, 1985

[cit58] Shehab M. , PhD thesis, University of Surrey, 2005

[cit59] Moulin E. , Busseron E. and Giuseppone N., Self Assembled Supramolecular Materials in Organic Electronics, in Supramolecular Materials for Opto-Electronics, ed. N. Koch, Royal Soc. Chemistry, London, 2015

[cit60] Mandalia H. C., Jain V. K. (2011). Adv. Anal. Chem..

[cit61] Chakraborty P., Chakrabarti C. L. (2008). Water, Air, Soil Pollut..

[cit62] Cox B. G., Hedwig G. R., Parker A. J., Watts D. W. (1974). Aust. J. Chem..

[cit63] Stevenson F. J. , Humus Chemistry: Genesis, Composition, Reactions, New York, John Wiley & Sons, 1994

[cit64] Humic Substances: Structures, Models and Functions, ed. E. A. Ghabbour and G. Davies, Royal Society of Chemistry, Cambridge, 2001

[cit65] Danil de Namor A. F., Sueros Velarde F. J., Casal A. R., Pugliese A., Goitia M. T., Montero M., Fraga Lopez F. (1997). J. Chem. Soc., Faraday Trans..

[cit66] Pugliese M. A., Goitia M. T., Montero M. E., Casal A. R., Danil de Namor A. F. (2006). Supramol. Chem..

[cit67] Danil de Namor A. F., Cleverley R. M., Zapata Ormachea M. L. (1998). Chem. Rev..

[cit68] Lima E. C., Hosseini-Bandegharaei A., Moreno-Piraján J. C., Anastopoulos I. (2019). J. Mol. Liq..

[cit69] Ghosal P. S., Gupta A. K. (2017). J. Mol. Liq..

[cit70] Liu Y., Xu H. (2007). Biochem. Eng. J..

[cit71] Liu Y., Liu Y. J. (2008). Sep. Purif. Technol..

[cit72] Liu Y. (2009). J. Chem. Eng. Data.

[cit73] Salvestrini S., Leone V., Iovino P., Canzano S. (2014). J. Chem. Thermodyn..

[cit74] Danil de Namor A. F., Khalife R. (2008). J. Phys. Chem. B.

[cit75] Danil de Namor A. F., Zvietcovich-Guerra J. A., Villanueva Salas J. A., Piro O. E., Webb O. A., El Gamouz A., Abou Hamdan W., Castellano E. E. (2015). RSC Adv..

[cit76] Aguado J., Arsuaga J. M., Arencibia A. (2005). Ind. Eng. Chem. Res..

[cit77] Antochshuck V., Olkhovyk O., Jaroniec M., Park I. S., Ryoo R. (2003). Langmuir.

[cit78] Hao N., Han L., Yang Y., Wang H., Webley P. A., Zhao D. (2010). Appl. Surf. Sci..

[cit79] Mercier L., Pinnavaia T. J. (1998). Environ. Sci. Technol..

[cit80] Olkhovyk O., Jarioniec M. (2005). Adsorption.

[cit81] Puanngam M., Unob F. (2008). J. Hazard. Mater..

[cit82] Schroden R. C., Al-Daous M., Sokolov S., Melde B. J., Lytle J. C., Stein A., Carbajo A. M. C., Fernández J. T., Rodríguez E. E. (2002). J. Mater. Chem..

[cit83] Shin S., Jang J. (2014). J. Chem. Soc., Chem. Commun..

[cit84] Showkat A. M., Zhang Y. P., Kim M. S., Gopalan A. I., Reddy K. R., Lee K. P. (2007). Bull. Korean Chem. Soc..

[cit85] Teng M., Wang H., Li F., Zhang B. (2011). J. Colloid Interface Sci..

[cit86] Wang X., Wang A. (2010). Sep. Sci. Technol..

