# Supplementary material for: A selective and easily recyclable dimer based on a calix[4]pyrrole derivative for the removal of mercury(ii) from water
Source: RSC Adv. 2020 Jan 16;10(6):3060–71. doi: 10.1039/c9ra09911e (PMC9048711; doi:10.1039/c9ra09911e)
Supplement: RA-010-C9RA09911E-s001 [file RA-010-C9RA09911E-s001.pdf]

This journal is © The Royal Society of Chemistry 2020

Supplementary information

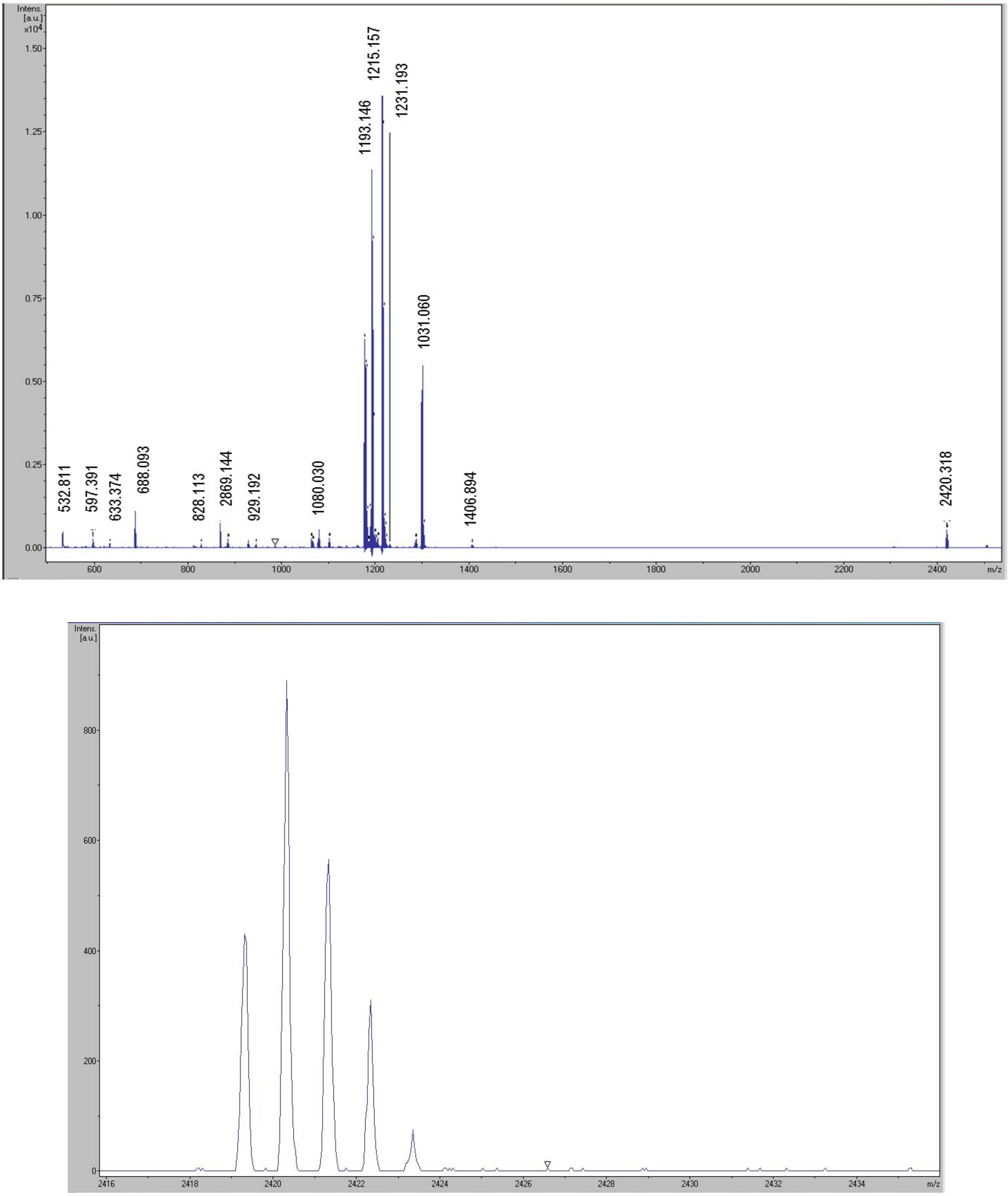

Fig. S1. Mass spectrum of the CPA dimer.

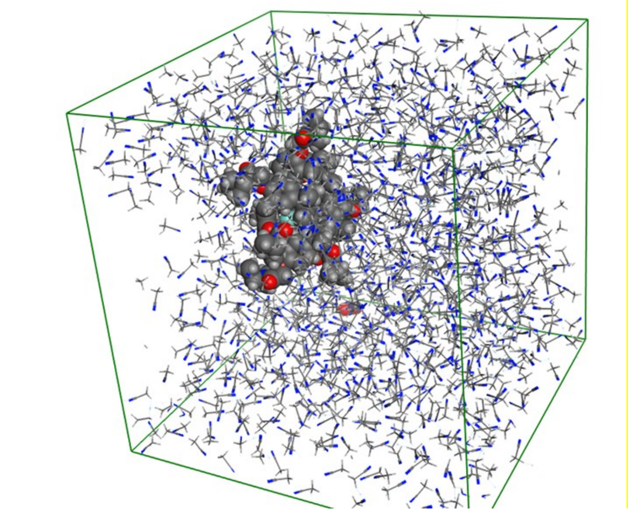

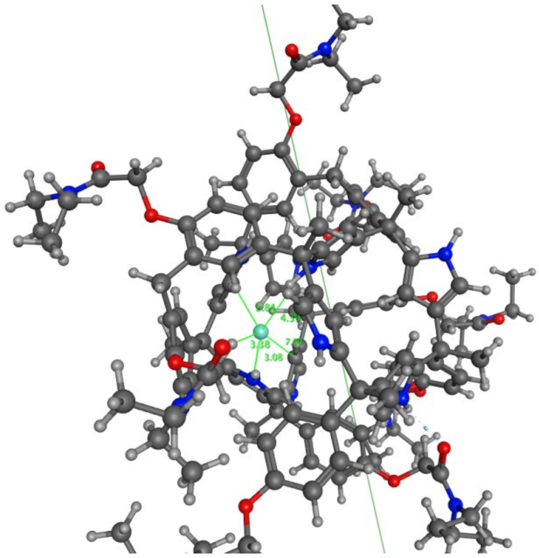

Hg (II) in contact with CPA dimer, NO_3 in solvent shell

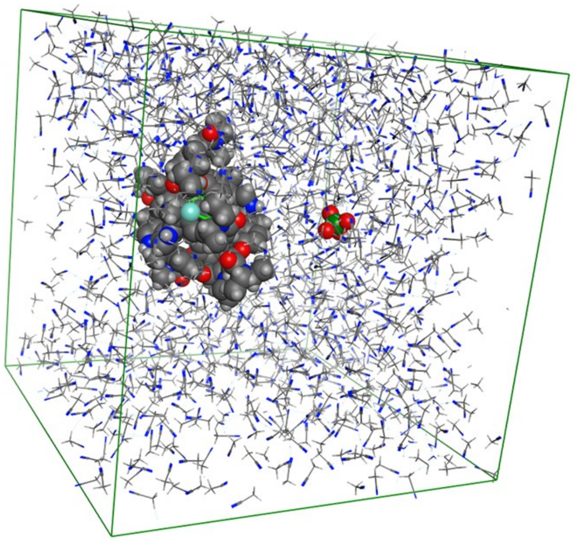

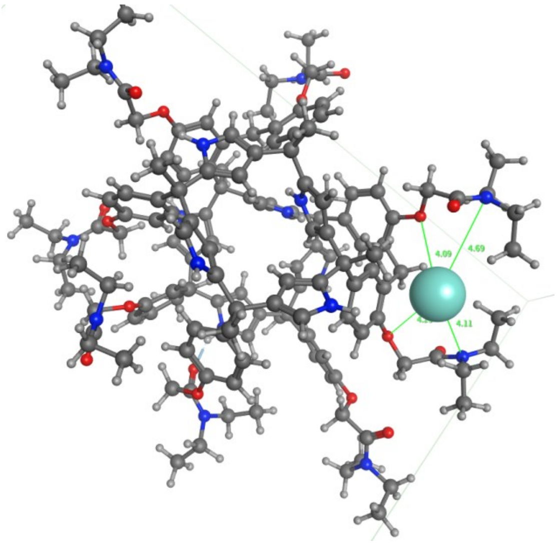

Hg (II) in contact with CPA dimer, ClO_4 in solvent shell

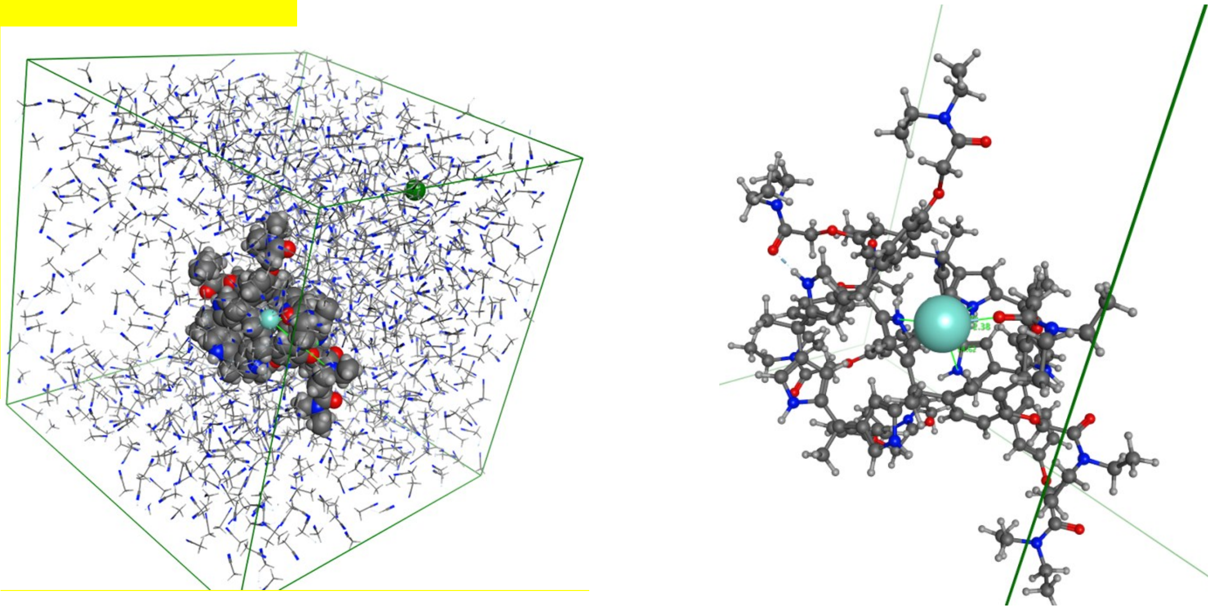

Hg (II) in contact with CPA dimer, Cl in solvent shell.

Fig. S2. Molecular Simulation dynamics of the dimer with Hg (II) involving different counter ions.
